# Anesthetic Management of a Pediatric Patient with Congenital Methemoglobinemia

**DOI:** 10.1155/2023/3474638

**Published:** 2023-01-04

**Authors:** Sung-Wook Choi, Elizabeth Putnam

**Affiliations:** ^1^Anesthesia and Perioperative Medicine, Pediatric Anesthesia, Medical University of South Carolina, 10 McClennan Banks Drive, Suite 2190, MSC 940, Charleston, South Carolina 29452, USA; ^2^Department of Pediatric Anesthesiology, University of Michigan Medicine, CS Mott Children's Hospital, 1500 E Medical Center Drive, Ann Arbor, Michigan 48109-5048, USA

## Abstract

A 15-year-old girl with congenital methemoglobinemia and no prior anesthetic history presented for the extraction of multiple impacted molars. Pulse oximetry values were expected to complicate the accurate monitoring of her oxygenation status in the perioperative period. An arterial line was placed for hemodynamic monitoring, arterial blood gas (ABG) analysis, and obtaining the methemoglobin (MetHgb) level and additional blood gases if clinically indicated. A continuous CO-oximeter was also used. Her intraoperative course was uneventful and she was discharged home on the same day of the procedure. This report reviews this rare condition and describes the monitoring methods utilized to assess her oxygen and methemoglobin levels, as well as the anesthetic techniques and pharmacologic agents employed. With appropriate intraoperative monitoring and careful drug selection, pediatric patients with this unusual condition can safely undergo general anesthesia.

## 1. Introduction

Congenital or hereditary methemoglobinemia is a rare condition which was first described in 1845 that prevents the reduction of the iron molecule contained in hemoglobin (Hb) from an oxidized state (Fe^3+^, ferric) to a reduced state (Fe^2+^, ferrous), resulting in decreased oxygen carrying capacity and tissue hypoxia [1]. To date, the literature on anesthesia has primarily described the perioperative management of adult patients with acquired methemoglobinemia or that of cyanotic patients who were later found to have previously undiagnosed congenital methemoglobinemia. We report the first case report of the perioperative management of a pediatric patient with known congenital methemoglobinemia undergoing general anesthesia.

## 2. Case Presentation

**Figure 1:**
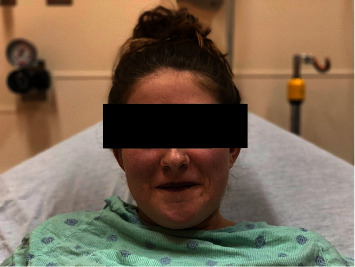
Our patient presented with the blue/purple shade of skin characteristic of congenital methemoglobinemia.

**Figure 2:**
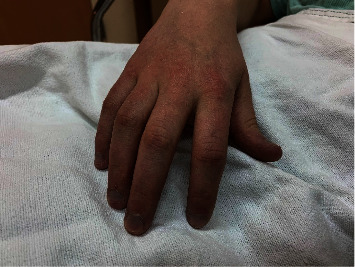
Cyanosis from congenital methemoglobinemia may also result in discoloration of the extremities and nails.

The patient was an otherwise healthy 15-year-old, 60 kg, Caucasian female (Figures 1 and 2), diagnosed with methemoglobinemia at birth after she was noted to have a “blue hue” without any accompanying symptoms. Her father, paternal grandmother, and paternal uncle had all been diagnosed with the same condition. Her functional status was high, as she was active in track and swimming while remaining asymptomatic. She was not taking any prescription medications. She presented for the extraction of multiple impacted molars under general anesthesia. Her preoperative hemoglobin and hematocrit values were 14.6 g/dL and 44.4%, respectively. Available outpatient MetHgb values varied between 3% and 15%. A preoperative interdisciplinary discussion was held before scheduling her procedure, and as the use of local anesthetics had the potential to cause methemoglobinemia, their use was avoided [2].

The patient's preinduction vital signs were as follows: blood pressure, 127/68; heart rate, 79; and respiration, 18 breaths/min. A nasal cannula was placed with an oxygen flow rate set at 2 L/min; a remifentanil and propofol infusion was started at 0.5 mcg/kg/hr and 100 mcg/kg/min, respectively, primarily in order to facilitate the placement of an arterial catheter without local infiltration, as well as to promote hemodynamic stability as a maintenance anesthetic. An initial arterial blood gas analysis, measured by a point-of-care GEM 3500, revealed a pH of 7.39, a PaO_2_ level of 110 mmHg, a PaCO_2_ level of 46 mmHg, an HCO_3_ level of 27.8 mmol/L, and a base deficit of 2.2 mmol/L. The patient was preoxygenated, and after an induction dose of propofol (150 mg) without neuromuscular blockade, the patient was intubated with a nasal RAE endotracheal tube on the first attempt, facilitated by the elective use of a flexible fiberoptic bronchoscope. Anesthesia was maintained with isoflurance delivered with oxygen and air, and remifentanil.

**Figure 3:**
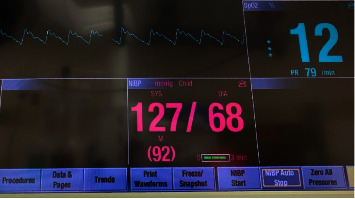
A standard pulse oximetry reading on room air.

**Figure 4:**
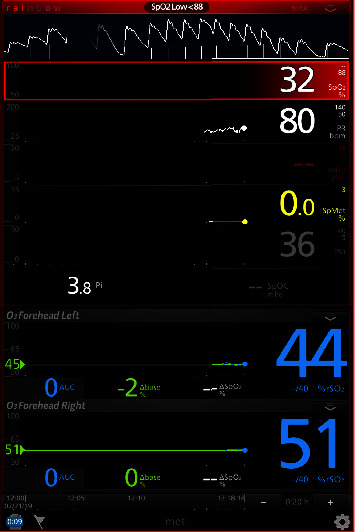
Our co-oximeter screen displaying pulse and cerebral oximetry values.

Pulse oximetry on room air revealed an oxygen saturation (SpO_2_) of 12%–35% (Figure 3), taken on all extremities. Cerebral oximetry, measured with an O_3_ near-infrared spectroscopy (NIRS) module, revealed values between 40% and55% (Figure 4). In an attempt to minimize arterial blood gas sampling, a Masimo Root monitor was utilized in conjunction with the Radical-7 Pulse CO-Oximeter (Masimo Inc., Irvine, CA) with the goal of measuring continuous MetHgb. As shown in Figure 4, cerebral oximetry values were visible, but the monitor was unable to detect the patient's methemoglobin level.

The patient received 4 mg each of dexamethasone and ondansetron as prophylaxis for postoperative nausea and vomiting, as well as 50 mcg of fentanyl for analgesia.

A second arterial blood gas at FiO_2_ level of 1.0 was drawn after intubation and sent to the hospital's main laboratory, which revealed a pH of 7.40, a PaO_2_ level of 452 mmHg, a PaCO_2_ level of 44 mmHg, an HCO_3_ level of 27.3 mmol/L, a base deficit of 2.1 mmol/L, and a serum methemoglobin level of 13.1%. End-tidal carbon dioxide (EtCO_2_) and NIRS values remained stable.

Approximately 15 minutes later, a repeat arterial blood gas at an FiO_2_ level of 0.7 revealed a pH of 7.38, a PaO_2_ level of 443 mmHg, a PaCO_2_ level of 43 mmHg, an HCO_3_ level of 25.2 mmol/L, a base deficit of 0.7 mmol/L, and a serum methemoglobin level of 13.2%. The case proceeded uneventfully, and the patient was extubated and taken to the post-anesthesia care unit (PACU). The total operative time was 40 minutes.

One final arterial blood gas was drawn in recovery with the patient being extubated and awake at 6 L/min of oxygen by facemask, demonstrating a pH of 7.39, a PaO_2_ level of 176 mmHg, a PaCO_2_ level of 40.5 mmHg, an HCO_3_ level of 24.3 mmol/L, a base deficit of 0.1 mmol/L, and a serum methemoglobin level of 13.8%. She was monitored for an additional four hours prior to being discharged home.

## 3. Discussion

Methemoglobinemia can result from various etiologies, including dietary, idiopathic, chemical, and toxicologic sources. It can also be congenital in nature and can be broadly classified into two categories, the first being methemoglobinemia due to an enzyme deficiency, specifically NADH reductase I deficiency, also referred to as cytochrome b5 reductase or diaphorase I deficiency. There are four known types of these disorders, all of which are autosomal recessive with respect to inheritance patterns [2]. Mutations of the alpha or beta globin chains can also result in an altered form of hemoglobin, which results in hemoglobin M disease, a condition that is inherited in an autosomal dominant fashion [3]. Our patient's family history suggested an autosomal dominant pattern of inheritance and in addition to her cyanosis present at birth, suggested that her diagnosis was hemoglobin M disease. However, her genetic workup had not been completed. The major characteristics of each subtype of congenital hemoglobinemia are summarized in Table 1.

Cyanosis is generally present when serum MetHgb levels reach approximately 5–15%. Patients with methemoglobinemia tend to present with symptoms such as dyspnea, headaches, dizziness, and/or confusion when MetHgb levels reach 30%. Increasing levels of neurologic decline, cardiovascular collapse, and death result from levels above 60–70%. Clinically, type II cytochrome b5 reductase deficiency is the most severe. While patients with other forms of congenital methemoglobinemia tend to remain asymptomatic with the exception of cyanosis, they are more susceptible to acquired forms of methemoglobinemia [4].

### 3.1. Anesthetic Considerations for Patients with Congenital Methemoglobinemia

The primary considerations in the perioperative care of patients with congenital methemoglobinemia relate to the assessment of oxygenation through the use of appropriate monitoring modalities and avoiding known triggering/oxidizing agents that could exacerbate their baseline methemoglobinemia.

Pulse oximetry devices rely on the difference in capacity for light absorption of oxygenated hemoglobin (HbO_2_) and deoxygenated hemoglobin (HHb). HbO_2_ absorbs more light in the infrared spectrum and HHb absorbs more red light. A finger pulse oximetry probe emits high-frequency light every 5–10 microseconds at two wavelengths: 660 nm (red) and 940 nm (infrared). A photo detector senses the pulsatile (AC) component of absorbed wavelengths, subtracts the nonpulsatile (DC) component, and generates a voltage proportional to the transmitted light. A microprocessor compares the absorption ratio of HbO_2_ and HHb by the two different wavelengths and converts this to a percentage using an algorithm of known values, which generates an SpO_2_ reading. The reading will be inaccurate if the pulsatile flow is very small (such as in hypotension or vasospasm), if the pulsatile flow is irregular (as in arrhythmias), or if nonstandard Hb components (such as CO or MetHgb) significantly interfere with wavelength absorbency.

MetHgb demonstrates light absorption at two peaks, 630 nm and 960 nm, i.e., absorption in each spectrum. As levels of MetHgb in the blood increase, equal absorption at these wavelengths corresponds to an SpO_2_ output by the microprocessor of 85% [5]. Even in the absence of increased methemoglobin levels, severe oxyhemoglobin desaturation can occur with even minimal durations of hypoventilation given these patients have only a modest amount of normal hemoglobin in addition to the left shift in their individual oxyhemoglobin dissociation curve, impairing oxygen delivery [6].

The utility of continuous CO-oximetry and cerebral oximetry has previously been described in the care of an adult with congenital methemoglobinemia and was utilized to guide the intraoperative management of increasing serum methemoglobin and ultimately methylene blue administration [7]. If such a device is unavailable, or reliable readings cannot be obtained, serial arterial blood gas measurements utilizing a laboratory-based CO-oximeter in conjunction with continuous end-tidal capnography can provide an adequate assessment of global oxygen delivery and tissue perfusion. Our Masimo device was calibrated and checked by a representative from the company, and the cause of the monitor's inability to measure MetHgb in our particular case remains unknown. The Masimo representative responded by suggesting that the blue discoloration of her fingers, or indeed, the low SpO_2_ measurement, may have contributed to this.

With respect to oxidizing agents, though many are known to trigger episodes of methemoglobinemia or an underlying condition, medications such as local anesthetics, antibiotics (dapsone), and nitrites (nitroglycerin/nitric oxide) are most commonly encountered by anesthesiologists in their clinical practice. Benzocaine and prilocaine account for the vast majority of local anesthetics associated with methemoglobinemia in the literature; however, bupivacaine's and lidocaine's roles in this regard are controversial and not definitively considered universally safe [8–10]. As discussed, patients with congenital methemoglobinemia, even if asymptomatic, are more likely to develop an acquired form when exposed to an oxidizing agent due to their decreased ability to reduce free radicals at the baseline, thus our reason to avoid the use of any local anesthetics in this patient's care [11, 12].

The mainstay of emergency therapy for acquired methemoglobinemia consists of intravenous administration of methylene blue, dosed at 1–2 mg/kg, given over 5–10 minutes, repeatable every half hour, up to a maximum of 7 mg/kg due to its own oxidant activity. It is generally indicated when MetHgb levels exceed 30% or earlier if the patients are symptomatic. Methylene blue works by activating nicotinamide adenine dinucleotide phosphate (NADPH) MetHgb reductase, ultimately reducing MetHgb to functional hemoglobin. In patients with glucose-6-P-dehydrogenase (G-6PD) deficiency, or chlorate poisoning, which inhibits G-6PD, methylene blue can actually exacerbate methemoglobinemia induced by intravascular hemolysis. In such cases, riboflavin can be effective in reducing MetHgb [12, 13].

Besides the enzyme-dependent pathways, there are bimolecular reactions that act to reduce MetHgb indirectly. For example, ascorbic acid reduces MetHgb in erythrocytes; it is oxidized to dehydroascorbic acid, which is then reduced by glutathione or DHA reductase, effectively transferring an electron to MetHgb. Thus, ascorbic acid can reduce oxidative stress and reduce MetHgb levels in patients with congenital methemoglobinemia after methylene blue treatment. Glutathione also plays a role in MetHgb reduction and is helpful in treating nitrite-induced methemoglobinemia [13].

## 4. Conclusion

Patients with congenital or inherited methemoglobinemia represent a challenge with respect to perioperative assessment of oxygenation and global tissue perfusion. The avoidance of potential oxidizing agents should be discussed, as well as the availability of emergency treatment modalities such as methylene blue or exchange transfusions, when applicable in the setting of an individual patient's specific disorder. When possible, the measurement of continuous MetHgb should be a consideration in their management, and when unavailable or not feasible, serial CO-oximetry via serial blood gas measurements in conjunction with other surrogate measures of oxygen delivery such as cerebral oximetry can be utilized to safely guide their care.

## Figures and Tables

**Table 1:** Congenital methemoglobinemia: pathophysiology and classification.

Disease subtypes	Pathophysiology	Features and prognosis
Cytochrome b5 reductase deficiency (type I)	Lack of cytochrome b5 reductase activity in erythrocytes only	Cyanosis develops at 6 to 9 months of age
		Usually asymptomatic, even with levels up to 40 percent
Cytochrome b5 reductase deficiency (type II)	Altered cytochrome b5 reductase function in various tissues, notable for a decrease in oxygen supply to the central nervous system	Associated with developmental delay, failure to thrive Clinically, the most severe variant and usually fatal in the first year of life
Cytochrome b5 reductase deficiency (type III)	Decrease in functional cytochrome b5 reductase activity in all cell types	Cyanotic at birth, otherwise asymptomatic
Cytochrome b5 reductase deficiency (type IV)	Decrease in functional cytochrome b5 reductase activity in all cell types	Similar to type I
Hemoglobin M disease	Mutations of the globin gene (alpha, beta, or rarely gamma). This results in an Fe^3+^-phenolate complex that resists reduction.	Characterized by chronic cyanosis, otherwise asymptomatic

## Data Availability

The data used to support this study are included within the article.
